# Monitoring the Athlete Match Response: Can External Load Variables Predict Post-match Acute and Residual Fatigue in Soccer? A Systematic Review with Meta-analysis

**DOI:** 10.1186/s40798-019-0219-7

**Published:** 2019-12-09

**Authors:** Karim Hader, Michael C. Rumpf, Maxime Hertzog, Liam P. Kilduff, Olivier Girard, Joao R. Silva

**Affiliations:** 1 National Sports Medicine Programme, Excellence in Football Project, Aspetar – Qatar Orthopaedic and Sports Medicine Hospital, P.O BOX 29222 Doha, Qatar; 2 Performance Department, Paris Saint-Germain F.C., Saint Germain-en-Laye, France; 3 Auckland University of Technology, Sport Performance Research Institute New Zealand, Auckland, New Zealand; 4 Footballscience.net, Roedermark, Germany; 5 A-STEM, College of Engineering, Swansea University, Swansea, UK; 6 Welsh Institute of Performance Science, Sport Wales, Cardiff, UK; 7 Murdoch Applied Sport Science Laboratory, Murdoch University, Perth, Western Australia; 8 Center of Research, Education, Innovation and Intervention in Sport (CIFI2D), Porto, Portugal

**Keywords:** External load, Time motion analysis, Monitoring, Fatigue, Recovery, Muscle damage, Perceptual responses, Neuromuscular performance

## Abstract

**Background:**

Monitoring athletes’ external load during a soccer match may be useful to predict post-match acute and residual fatigue. This estimation would allow individual adjustments to training programs to minimize injury risk, improve well-being, and restore players’ physical performance and inform the recovery process.

**Methods:**

Using a systematic review and meta-analysis of the literature, the aim is to determine which monitoring variables would be the strongest predictors of acute (immediately) and residual (up to 72 h) fatigue states in soccer. PubMed, SPORTDiscus, and Web of Science databases were searched (until September 2018). Studies concurrently examining soccer match-related external load metrics and subjective and/or objective measures were selected to determine pooled correlations ($\overline{r}$) with confidence intervals (CI). The quality and strength of the findings of each study were evaluated to identify overall levels of evidence.

**Results:**

Eleven studies were included (*n* = 165 athletes). Acute ($\overline{r}$ = 0.67; 95% CI = [0.40, 0.94]) and residual (24 h post-match, $\overline{r}$ = 0.54; 95% CI = [0.35, 0.65]) changes in muscle damage markers and countermovement jump peak power output (CMJ_PPO_) were, with moderate to strong evidence, largely correlated with running distance above 5.5 m s^−1^. No other external load metric was largely correlated with both biochemical and neuromuscular markers. For every 100-m run above 5.5 m·s^−1^, CK activity measured 24 h post-match increased by 30% and CMJ_PPO_ decreased by 0.5%. Conversely, the total distance covered did not present any evidence of a clear relationship with any fatigue-related marker at any time-point.

**Conclusions:**

Running distance above 5.5 m·s^−1^ represents the most sensitive monitoring variable characterizing biochemical and neuromuscular responses, at least when assessed during the initial 24 h (not at 48 h/72 h) post-match recovery period. In addition, total distance covered is not sensitive enough to inform decision-making during the fatigue monitoring process.

## Key Points

- The running distance covered above 5.5 m·s^−1^ represents the most sensitive monitoring variable estimating post-match (up to + 24 h but not at 48–72 h) changes in biochemical and neuromuscular responses.
- Total distance covered may represent the less sensitive variable to monitor.
- For every 100-m run above 5.5 m·s^−1^ during match-play, creatine kinase activity measured 24 h post-match may increase by 30% and CMJ peak power output decrease by 0.5%.

## Background

Soccer is considered a high-intensity intermittent sport with an unprecedented increase (up to 50%) in high-impulsive actions (e.g., number of high accelerations, sprint distance covered) occurring during match-play reported over the last decade [1]. Monitoring players’ responses (e.g., physiological and perceptual) to a soccer match is paramount to prescribe the optimal training dose at an individual level, minimize injuries, and restore physical performance for subsequent training and competition [2, 3]. During the past decade, there has been a substantial development of computer-aided tracking technology (e.g., multiple camera semi-automatic systems) for the examination of players’ external load [4] (i.e., activity performed such as total distance covered or the number of accelerations [5]) during training and match-play. These sophisticated systems are now capable of providing detailed analysis of external load demands, which allows individualized performance profiling of players to tailor training programs [4]. The emergence of manufacturer-specific algorithms has prompted the development of some recent parameters (e.g., player load), expressed in absolute or relative terms, yet with a lack of validity and reliability for many of them such as metabolic power and its derivatives [6, 7]. Despite substantial technological advancements, there is a lack of consensus for selecting the most appropriate parameters for quantifying the short-term dose-response relationship and precisely informing on the “stress” experience by each individual player in elite soccer [8].

Soccer match-play is a stressor for various physiological (e.g., musculoskeletal, immunological, metabolic) regulatory systems [9, 10]. This stress results in acute (i.e., less than 3 h post-match) and residual (still evident up to 72 h post-match) fatigue-induced impairments commonly characterized by neuro-mechanical alterations (e.g., decrease in maximal force production capacity) [10–12], perturbations in the biochemical milieu (e.g., increase in creatine kinase levels) [10, 13, 14] and in the psychometric state [10, 15]. While several factors (e.g., genotype and phenotype) [16] likely influence internal load experienced by each individual player, specific external load metrics (e.g., acceleration variables) may estimate match-related players’ fatigue status. It is thought that locomotor activities requiring repeated eccentric muscle contractions (e.g., acceleration and deceleration patterns, high-speed running distance) [17] can explain the aforementioned metabolic, physical, and psychometric disturbances [13, 15, 18, 19] and, in turn, the potential injury causation [19, 20]. However, the relationship between match external load metrics and markers of post-soccer-match fatigue remains unclear with conflicting results in the literature. For example, the extent of acute muscle damage was correlated with high-intensity (HI) running distance (> 4 m s^−1^) with either small (*r* = 0.24) [21] or very large magnitudes (*r* = 0.92 )[22]. Similar differences in magnitude exist between the extent of residual muscle damage (i.e., 48 h following the match) and high decelerations (*r* = 0.19 and 0.71) [12, 23]. Therefore, the lack of consensus regarding the effectiveness/preferred external load metrics to monitor players’ physiological and biochemical responses to a soccer match implies a need for a systematic review with meta-analysis. This may identify the most sensitive monitoring variables associated with post-match acute and residual fatigue-related markers. This information may allow informed decisions from a fatigue monitoring standpoint as well from a return to play and performance management perspective.

Therefore, using a systematic review and meta-analysis of the literature, the aim is to determine which external load metrics during a soccer match-play more effectively reflect the acute and residual changes in post-match muscle damage and neuromuscular and perceptual responses.

## Methods

### Literature Search Strategy

The systematic review with meta-analysis was conducted in accordance with the recommendations defined in the Preferred Reporting Items for Systematic Reviews and Meta-Analyses statement (PRISMA) and the Population-Intervention-Comparators-Outcomes-Study design (PICOS) approach [24]. The literature search was computerized using PubMed, SPORTDiscus, and Web of Science databases, until the end of September 2018. The complete Boolean search strategy is presented in Table 1. No sex restriction was imposed during the search stage. The reference lists of all articles were examined to identify further eligible studies. Papers published in the *epub ahead of print* within the abovementioned time frame were also considered.

**Table 1:** Database search strategy

1.“soccer match” OR “football match”	
2.“locomotor activity” OR “match activity” OR “match load” OR “external load” OR “monitoring”	
3.“muscle damage” OR “creatine kinase” OR “biochemical markers”	
4.“muscle performance” OR “jump” OR “strength” OR “power” OR “neuromuscular”	
5.“fatigue” OR “recovery” OR “perceptual” OR “soreness” OR “perceived exertion” OR “internal load” OR “psychometric”	
(1 AND 2 AND 3) OR (1 AND 2 AND 4) OR (1 AND 2 AND 5)	

The PICOS approach of this investigation can be detailed as follows: **Population**: soccer players. **Intervention**: official or friendly soccer match without extra time. **Comparators**: players’ soccer match-related external load metrics (e.g., players’ running distance above speed thresholds). **Outcomes variables**: the dependent variables are the acute (i.e., immediately) and residual (1, 2, and 3 days following the match) changes in biochemical, neuromuscular, and perceptual measures [12, 25, 26]. **Study design**: observational studies with a before-after intervention (i.e., a soccer match).

### Study Selection and Quality Assessment

A second phase consisted of applying selected inclusion criteria. These inclusion criteria were as follows:

1. The study was an original research, published in the English language in a peer-review journal.
2. The study population was soccer players.
3. The intervention was an on-field soccer match.
4. The time-motion analysis of locomotor-related activities was reported.
5. Measures of post-match muscular performance or markers of muscle damage or psychometric state were presented.
6. A correlation coefficient reflecting the relationship between one or more external load metrics and post-match fatigue-related markers was reported, or information needed to compute this coefficient was mentioned or available on a supplement file or was obtained from the author(s) of the study.

Attempts were made to contact the authors of the selected articles to request missing data. All authors were given 3 weeks to provide that data. After this period, the studies were assessed for risk of bias using an adapted version of a published scoring system [27]. Ten criteria were determined using the National Heart Blood Institute (NIH) guidelines for qualitative evaluation of observational cohort and cross-sectional studies and before-after (pre-post) studies with no control group. In addition, other versions of currently established scales used in sports sciences (e.g., Delphi and PEDro Scale, Newcastle – Ottawa quality assessment scale, Downs and Black) were considered. The quality assessment was based on the reporting of study methods and results with answer categories being “yes,” “partial,” and “no” (Table 2).

**Table 2:** Quality assessment criteria

No.	Items	Scoring		
		0	1	2
1	Was the main question or objective clearly described?	No		Yes
2	Was the study population clearly specified and defined (age, gender, training status, stated inclusion/exclusion criteria)?	No	Partly	Yes
3	Was the sample size justified?	No		Yes
4	Main measured variables clearly described in the Introduction or Method section?	No	Partly	Yes
5	Locomotor activity variables clearly defined (thresholds, ranges).	No	Partly	Yes
6	Were the validity and reliability of the main variables measurements discussed?	No	Partly	Yes
7	Were the methods (included the statistical methods) sufficiently described to enable the study replication?	No	Partly	Yes
8	Does the study provide estimates of the random variability in the data for the main outcomes (i.e. confidence intervals, standard deviations)?	No	Partly	Yes
9	Were all the tested associations reported?	No		Yes
10	Were the study limitations discussed?	No	Partly	Yes

Summary scores (ranging from 0 to 1) were calculated as follows:

Summary score = [(number of ‘yes’ × 2) + (number of ‘partial’ × 1)] / (number of criteria × 2) (1)

Studies were then classified as high (≥ 0.75), moderate (0.50–0.75), or poor methodological quality (< 0.50) [27].

### Independent Variables

The independent variables consisted of external load metrics related to soccer match-play. They are presently described by Gray’s classification [2] using three distinct levels:

- Level 1: Typical distances covered in different running speeds
- Level 2: All events related to changes in running speed: accelerations, decelerations, and changes of directions
- Level 3: All events derived from the inertial sensors/accelerometers such as impacts above gravitational force thresholds

However, in each individual selected study, external load variables may have been presented with specific speed thresholds and, in turn, defined differently. Sprinting pattern, for example, has been defined as running speed above different thresholds: (i) 5 m s^−1^ [22], (ii) 5.5 m s^−1^ [12], (iii) 5.8 m s^−1^ [23], and (iv) 7 m s^−1^ [21]. Consequently, the independent variables were grouped by common zones based on thresholds used by practitioners on the field in elite soccer [8]:

- High-intensity running (HIR): running speed greater than ~ 4 m s^−1^.
- Very high-intensity running (VHIR): running speed greater than 5 to 5.5 m s^−1^
- Sprint: running speed greater than 7 m s^−1^.
- Moderate to high-intensity acceleration: acceleration greater or equal to + 2 m·s^−2^.
- High-intensity acceleration: acceleration greater or equal to + 3 m s^−2^.
- High-intensity deceleration: a deceleration lower or equal to − 3 m s^−2^.
- Moderate- to high-intensity deceleration: a deceleration lower or equal to − 2 m s^−2^.
- High-intensity impact: impact greater than 7 G (gravitational force). The impact is the instantaneous rate of acceleration and deceleration in the three axes, measured by the integrated accelerometer.
- High change of direction: change of direction with a high-intensity deceleration.

### Dependent Variables

The dependent variables extracted from the selected studies were systematically reviewed and grouped into three categories: biochemical, neuromuscular, and perceptual measures. Changes in the biochemical milieu were assessed through endocrine, immunological, and muscle damage markers (Table 3). Endocrine alterations consisted of changes in testosterone and cortisol concentrations. Changes in immunological markers were assessed by leucocytes counts. Changes in muscle damage markers consisted of measures of intracellular enzyme activity (creatine kinase and lactate dehydrogenase, CK and LDH, respectively) and in circulating concentrations of Myoglobin (Mb). The neuromuscular function was assessed by (i) maximal voluntary contractions using isokinetic dynamometers to measure several force-related variables (e.g., peak torque achieved by different lower-limbs muscle groups under maximal isometric; MVIC), concentric and eccentric contractions, (ii) vertical jump performance using force plates and/or optical timing systems (e.g., jump peak power output during a countermovement, CMJ_PPO_). The perceptual responses were mainly based on lower limb delayed onset of muscle soreness (DOMS), rate of perceived exertion (RPE), the perceived recovery (TQR), and the brief assessment of mood (BAM+) measures. DOMS was assessed with visual analog scales in response or not to a “conditioning” stimulus (e.g., squatting). Other scales were used to quantify RPE, TQR, and BAM+.

**Table 3:** Characteristics of the selected studies

Studies	Players details (level, *n*, age)	Tracking systems	External load metrics examined	Fatigue-related dependent variables investigated	Quality assessment score
Aquino et al., [28]	Elite/18/15.6 ± 0.4	Video analysis "tracking software DVIDEOW"	Medium running distance (8.1–13 km h^−1^); HI running distance (13.1–18 km h^−1^); Sprinting distance (> 18 km h^−1^); HI activity distance (> 13 km h^−1^); TD; Number of sprints.	Pre- to post-match % change in CK concentration Pre- to post-match % change in LDH concentration	0.65
De Hoyo et al., [23]	Elite/15/18 ± 1	15-Hz GPS with 100-Hz accelerometer	Medium intensity (14–17.9 km h^−1^), HI (18-20.9 km h^−1^) and sprinting (> 21 km h^−1^) distances; High-speed (> 14 km h^−1^) and very high-speed running (> 18 km h^−1^); number of medium to high (> 2 m/s^2^) and high (> 3 m/s^2^) accelerations; number of medium to high (< − 2 m/s^2^) and high decelerations (< − 3 m/s^2^); TD; Impacts (> 7.1G)	Pre to post-match changes at ~ 30 min, G + 24H and G + 48H in: CK concentration Average concentric and eccentric force during CMJ CMJ height	0.70
Draganidis et al., [21]	Semi-professional/20/ 22.6 ± 1.5	5-Hz GPS with 100-Hz accelerometer	HI running (>14.5 km h^−1^); VHI running (>19.8 km h^−1^) and sprinting (>25.2 km h^−1^); number of low, medium and HI accelerations (1–2 m/s^2^, 2–3 m/s^2^, > 3 m/s^2^); Number of low, medium and HI decelerations (− 1 to − 2 m/s^2^, − 2 to − 3 m/s^2^, < − 3 m/s^2^); TD	Pre to G + 24H, G + 48H and G + 72H changes in: CK concentration Leucocytes count Concentric and eccentric isokinetic peak torque at 60°/s and 180°/s MVIC of KE and KF muscles DOMS	0.80
Nedelec et al., [25]	Professional/14/ 21.8 ± 3.2	Video analysis	High acceleration and deceleration <5 m High acceleration and deceleration >5 m HI running Hard changes of direction	Pre to G + 24H, G + 48H and G + 72H post-match changes in: CMJ performance MVIC of the hamstring with knee flexed at 90° and 150° KF muscle soreness	0.65
Rampinini et al., [29]	Professional/20/19 ± 1	Video-computerized, semi-automatic match analysis	TD HI running (> 15 km h^−1^)	Pre to immediately, G + 24H and G + 48H % changes in: Maximal voluntary activation (%VA) Normalized Root mean square of vastus lateralis muscle EMG signal during MVIC. Peak torque with 1-ms, 10-ms interval and 100-ms interval stimulations	0.70
Romagnoli et al., [30]	Professional/20/17- 20	Video-computerized, semi-automatic match analysis	TD Low-intensity running distance (< 15 km h^−1^) HI running distance (> 15 km h^−1^) VHI running distance (> 20 km h^−1^)	Pre to 30 min, G + 24H and G + 48H changes in: White blood cells count Cortisol and Testosterone concentrations Creatine kinase concentration	0.40
Russell et al., [12]	Professional/15/ 20 ± 1	10-Hz GPS units	Raw and normalized (per min) TD Raw and normalized (per min) HI running distance (> 19.8 km h^−1^) Raw and normalized (per min) total number of: sprints (> 19.8 km h^−1^), high accelerations, high decelerations, HI impacts Total number of accelerations, decelerations and impacts.	Pre to G + 24H and G + 48H changes in: CK concentration CMJ PPO	0.80
Scott et al., [31]	Professional/15/26 ± 4	video-computerized, semi-automatic match analysis	TD, HI running (> 19.8 km h^−1^) and Sprinting (> 25.2 km h^−1^): distances and occurrences	Pre to G + 48H changes in CK concentration	0.75
Shearer et al., [32]	Elite/11/20 ± 1	10-Hz GPS units	Raw and normalized (per min) TD Raw and normalized (per min) HI running distance (> 19.8 km h^−1^) Raw and normalized (per min) total number of sprints (> 19.8 km h^−1^)	Pre to G + 24H and G + 48H changes in: CK concentration CMJ PPO Brief assessment of mood	0.75
Thorpe et al., [22]	Semi-professional/7/25 ± 6	1-Hz GPS unit with 100-Hz accelerometer	Walking distance (0–6 km h^−1^); jogging distance (6–8 km h^−1^); low-speed running (8–12 km h^−1^); moderate-speed running (12–15 km h^−1^); Fast speed running (15–18 km h^−1^); sprinting distance (>18 km h^−1^); HI activity (> 15 km h^−1^); total number of sprints.	Pre to post-match changes in: CK concentration Myoglobin concentration Cortisol and Testosterone concentrations	0.70
Varley et al., [33]	Professional/10/27 ± 3	Video-computerized, semi-automatic match analysis	TD, sprint distance (> 25.2 km h^−1^), total number of: sprints), high accelerations, high decelerations.	Pre to immediately, G + 48H and G + 72H changes in: CK concentration CMJ height Muscle fatigue and wellness	0.85

*n*: number of players, *TD*: total distance, *KE*: knee extension, KF: knee flexion, CMJ: countermovement jump, PPO: peak power output, PT: peak torque, DOMS: delayed onset muscle soreness, HI: high intensity, VHI: very high intensity, MVIC: maximal voluntary isometric contraction

### Analysis and Interpretation of Results

To determine the relationship between match-related external load variables and post-match fatigue-related measurements, each relationship from an individual study was rated according to its direction (positive, negative, no relationship) and its magnitude as determined from reported correlations. The criteria adopted to categorize magnitudes of correlations (r) were as follows: ≤ 0.1, trivial; > 0.1–0.3, small; > 0.3–0.5, moderate; > 0.5–0.7, large; > 0.7–0.9, very large; and > 0.9–1.0, almost perfect [34]. Individual relationships were then summed and rated according to the predetermined levels of evidence adapted from Van Tulder et al. [35] recommendations:

*➢ Strong evidence*: consistently identified in two or more studies, which presented low heterogeneity (*I*^2^ < 30%) and including a minimum of two high-quality studies.

*➢ Moderate evidence*: consistently identified in two or more studies, which presented low heterogeneity (*I*^2^ < 30%) and including at least one high-quality study.

*➢ Limited evidence*: identified in one high-quality study, or multiple low- to moderate-quality studies that may not present low heterogeneity (*I*^2^ < 30%).

*➢ Conflicting evidence*: inconsistency in two or more studies where half of the studies are in agreement and the other half conflicting.

*➢ No evidence*: pooled results that are insignificant and derived from multiple moderately to substantially heterogeneous studies (*I*^2^ > 30%).

“Inconsistency” refers to a lack of similarity for correlation coefficients across studies. Study results are considered consistent when direction, magnitude, and statistical significance are sufficiently similar to lead to the same conclusions [36]. Consistency in direction is defined as 75% or more of the studies showing either a positive or negative correlation. Consistency in magnitude is defined as 75% or more of the studies showing an important or unimportant relationship [36]. Heterogeneity between studies was assessed using the *I*^2^ statistic, where an *I*^2^ of 30% or less is considered to indicate low heterogeneity and the cutoffs of 30% < I^2^ < 50% and *I*^2^ > 50% are indicative of moderate and substantial heterogeneity, respectively [37]. *I*^2^ describes the percentage of total variation across studies that is due to heterogeneity rather than chance and seeks to determine whether there are genuine differences underlying the results of the studies (heterogeneity), or whether the variation in findings is compatible with chance alone (homogeneity) [37]. Heterogeneity between studies was also assessed by the chi-squared test [36].

The meta-analysis was processed in two consecutive phases. Some studies investigating the changes in muscle damage determined the activity of different serum skeletal muscle proteins (e.g., CK and LDH). As various markers are believed to provide a composite picture of muscle damage status, using more than one marker has been recommended [38]. Consequently, the correlation coefficients, associating two muscle damage markers (e.g., CK and LDH) with identical external load metric (e.g., HI running distance) were combined in this first step to obtain a single within-study correlation coefficient. The specific relationships (e.g., HI running distance vs. muscle damage markers) assessed in several selected studies were then meta-analyzed between studies during the second phase.

The meta-analytic procedure was conducted with StatsDirect software (v 2.8.0, StatsDirect, Cheshire, UK) package allowing to calculate pooled correlation coefficients ($\overline{r}$) with three different methods: (i) Hedges and Olkins random-effects method [39], (ii) Hedges and Olkins fixed-effects method and, (iii) Hunter and Schmidt random-effects method [40]. The latter method systematically presented equivalent or the lowest pooled correlation coefficient. Therefore, to minimize overestimation bias, it was decided to consider the results from Hunter and Schmidt method only (Tables 4 and 5). Moreover, for either a small number of studies (less than 30) or a heterogeneous set of studies, the least biased estimate of the true population correlation is believed to be provided by Hunter and Schmidt method [41]. Pooled correlation coefficients are presented with 95% of confidence limits/intervals (CL/CI). Finally, once the strongest predictor has been determined, the studies investigating this external variable were selected and the authors were contacted to obtain individual data. This allowed us to provide a relationship between this predictor and fatigue-related markers (percentage change in CMJ_PPO_ and CK, Fig. 2).

**Table 4:** Summary of findings from meta-analysis describing the relationship between external load metrics of level 1 [3] and fatigue-related markers

Predictors	Fatigue-related markers	Time	ṝ	Lower CL	Upper CL	Number of studies/correlations	Evidence	*n*	*I*^2^	chi^2^	*p*
TD	Muscle damage	Post	0.36	0.05	0.67	5/3	Limited	48	66	2.95	0.09
		G + 24H	0.36	0.19	0.53	5/6	Limited	87	0	4.73	0.45
		G + 48H	0.23	0.05	0.42	6/7	Moderate	97	0	4.81	0.44
		G + 72H	− 0.02			1/1	No	10			
	CMJ_PPO_	G + 24H	− 0.16	− 0.47	0.15	2/5	Conflicted	67	41	7.83	0.10
		G + 48H	0.09	− 0.08	0.27	2/5	Moderate	67	0	2.46	0.65
	CMJ performance	G + 48H	0.04			1/1	No	10			
		G + 72H	− 0.06			1/1	No	10			
	Leucocytes count	G + 24H	− 0.10			2/1	No	39			
		G + 48H	0.21			2/1	No	39			
		G + 72H	0.09			2/1	No	39			
	Cortisol	Post	NS			1/0	No				
		G + 24H	0.50			1/1	Limited	20			
		G + 48H	0.52			1/1	Limited	20			
	Testosterone	Post	NS			1/1	No	20			
		G + 24H	NS			1/1	No	20			
		G + 48H	NS			1/1	No	20			
	PT 1 Hz	Post	− 0.19			1/1	No	19			
	PT 10 Hz	Post	− 0.20			1/1	No	19			
	PT 100 Hz	Post	− 0.17			1/1	No	19			
	DOMS	G + 24H	− 0.04			1/1	No	20			
		G + 48H	0.06			1/1	No	20			
		G + 72H	0.04			1/1	No	20			
	PT Concentric KE	G + 24H	− 0.68			1/1	Limited	20			
		G + 48H	0.43			1/1	Limited	20			
		G + 72H	0.42			1/1	Limited	20			
	PT Concentric KF	G + 24H	− 0.76			1/1	Limited	20			
		G + 48H	0.65			1/1	Limited	20			
		G + 72H	0.58			1/1	Limited	20			
	PT Eccentric KE	G + 24H	− 0.59			1/1	Limited	20			
		G + 48H	0.40			1/1	Limited	20			
		G + 72H	0.44			1/1	Limited	20			
	PT Eccentric KF	G + 24H	− 0.82			1/1	Limited	20			
		G + 48H	0.58			1/1	Limited	20			
		G + 72H	0.56			1/1	Limited	20			
	BAM+	G + 24H	0.09			1/1	No	35			
		G + 48H	0.14			1/1	No	35			
TD/min	Muscle damage	G + 24H	0.13	0.05	0.26	2/2	Limited	67			
		G + 48H	− 0.06	0.00	− 0.11	2/2	Limited	67			
	CMJ_PPO_	G + 24H	− 0.03	− 0.05	− 0.01	2/2	Limited	67			
		G + 48H	− 0.11	− 0.11	− 0.10	2/2	Limited	67			
	BAM+	G + 24H	− 0.47			1/1	Limited	35			
		G + 48H	− 0.51			1/1	Limited	35			
HIR	Muscle damage	Post	0.55	0.33	0.78	5/3	Moderate	45	59	3.40	0.18
		G + 24H	0.37			3/ 1	Limited	20			
		G + 48H	0.37	0.21	0.53	3/2	Limited	35	0	0.57	0.45
	MVIC Dom	G + 24H	− 0.55			2/1	Limited	20			
		G + 48H	0.43			2/1	Limited	20			
		G + 72H	0.46			2/1	Limited	20			
	DOMS	G + 24H	− 0.03			2/1	No	34			
		G + 48H	0.00			2/1	No	34			
		G + 72H	0.04			2/1	No	34			
	Leucocytes count	G + 24H	− 0.11			2/1	No	39			
		G + 48H	0.21			2/1	No	39			
	Cortisol	Post	NS			2/0	No				
		G + 24H	NS			1/0	No				
		G + 48H	NS			1/0	No				
	Testosterone	Post	NS			2/0	No				
		G + 24H	NS			1/0	No				
		G + 48H	NS			1/0	No				
	PT 1 Hz	Post	− 0.22			1/1	No	19			
	PT 10 Hz	Post	− 0.24			1/1	No	19			
	PT 100 Hz	Post	− 0.20			1/1	No	19			
	PT Concentric KE	G + 24H	− 0.69			1/1	Limited	20			
		G + 48H	0.46			1/1	Limited	20			
		G + 72H	0.45			1/1	Limited	20			
	PT Concentric KF	G + 24H	− 0.77			1/1	Limited	20			
		G + 48H	0.68			1/1	Limited	20			
		G + 72H	0.59			1/1	Limited	20			
	PT Eccentric KE	G + 24H	− 0.59			1/1	Limited	20			
		G + 48H	0.43			1/1	Limited	20			
		G + 72H	0.46			1/1	Limited	20			
	PT Eccentric KF	G + 24H	− 0.83			1/1	Limited	20			
		G + 48H	0.60			1/1	Limited	20			
		G + 72H	0.58			1/1	Limited	20			
VHIR	Muscle damage	Post	0.67	0.40	0.94	5/3	Moderate	45	77	8	0.02
		G + 24H	0.54	0.35	0.65	5/6	Strong	87	0	2.80	0.73
		G + 48H	0.36	0.19	0.53	5/7	Moderate	87	0	4.55	0.47
	CMJ_PPO_	G + 24H	− 0.52	− 0.64	− 0.40	2/5	Strong	67	0	2.21	0.70
		G + 48H	− 0.25	− 0.48	− 0.02	2/5	Limited	67	0	4.63	0.33
	MVIC Dom	G + 24H	− 0.57			1/1	Limited	20			
		G + 48H	0.45			1/1	Limited	20			
		G + 72H	0.43			1/1	Limited	20			
	Leucocytes count	G + 24H	− 0.12			2/1	No	39			
		G + 48H	0.19			2/1	No	39			
	BAM+	G + 24H	− 0.21			1/1	No	35			
		G + 48H	− 0.17			1/1	No	35			
	Cortisol	Post	NS			2/0	No				
		G + 24H	NS			1/0	No				
		G + 48H	NS			1/0	No				
	Testosterone	Post	NS			2/0	No				
		G + 24H	NS			1/0	No				
		G + 48H	NS			1/0	No				
	CMJ performance	Post	NS			1/0	No				
		G + 24H	NS			1/0	No				
		G + 48H	NS			1/0	No				
	DOMS	G + 24H	0.07			1/1	No	20			
		G + 48H	0.07			1/1	No	20			
		G + 72H	− 0.10			1/1	No	20			
	PT Concentric KE	G + 24H	− 0.69			1/1	Limited	20			
		G + 48H	0.51			1/1	Limited	20			
		G + 72H	0.51			1/1	Limited	20			
	PT Concentric KF	G + 24H	− 0.78			1/1	Limited	20			
		G + 48H	0.72			1/1	Limited	20			
		G + 72H	0.61			1/1	Limited	20			
	PT Eccentric KE	G + 24H	− 0.59			1/1	Limited	20			
		G + 48H	0.51			1/1	Limited	20			
		G + 72H	0.48			1/1	Limited	20			
	PT Eccentric KF	G + 24H	− 0.83			1/1	Limited	20			
		G + 48H	0.63			1/1	Limited	20			
		G + 72H	0.62			1/1	Limited	20			
VHIR dist/min	Muscle damage	G + 24H	0.37	0.11	0.55	2/5	Limited	67			
		G + 48H	0.13	0.05	0.31	2/5	Limited	67			
	CMJ_PPO_	G + 24H	− 0.22	− 0.48	− 0.03	2/5	Limited	67			
		G + 48H	− 0.25	− 0.53	0.05	2/5	Limited	67			
	BAM+	G + 24H	− 0.44			1/1	Limited	35			
		G + 48H	− 0.40			1/1	Limited	35			
VHIR runs/min	Muscle damage	G + 24H	0.24	0.06	0.42	2/5	Limited	67			
		G + 48H	0.11	− 0.05	0.29	2/5	Limited	67			
	CMJ peak power	G + 24H	− 0.22	− 0.49	− 0.05	2/5	Limited	67			
		G + 48H	− 0.20	− 0.41	− 0.02	2/5	Limited	67			
	Cortisol	Post	NS			1/1	No				
	Testosterone	Post	NS			1/1	No				
	BAM+	G + 24H	− 0.55			1/1	Limited	35			
		G + 48H	− 0.49			1/1	Limited	35			
Sprint	Muscle damage	Post	0.12			2/1	Limited	35			
		G + 24H	0.40			2/1	Limited	35			
		G + 48H	0.38			3/2	Limited	45			
		G + 72H	− 0.02			1/1	Limited	10			
	MVIC	G + 24H	− 0.51			2/1	Limited	20			
		G + 48H	0.35			2/1	Limited	20			
		G + 72H	0.35			2/1	Limited	20			
	Leucocyte count	G + 24H	− 0.10			1/1	Limited	20			
		G + 48H	0.17			1/1	Limited	20			
		G + 72H	− 0.23			1/1	Limited	20			
	DOMS	G + 24H	0.31			2/2	Limited	20			
		G + 48H	0.05			2/1	Limited	20			
		G + 72H	− 0.37			2/1	Limited	20			
	PT Concentric KE	G + 24H	− 0.58			1/1	Limited	20			
		G + 48H	0.55			1/1	Limited	20			
		G + 72H	0.58			1/1	Limited	20			
	PT Concentric KF	G + 24H	− 0.70			1/1	Limited	20			
		G + 48H	0.70			1/1	Limited	20			
		G + 72H	0.56			1/1	Limited	20			
	PT Eccentric KE	G + 24H	− 0.45			1/1	Limited	20			
		G + 48H	0.64			1/1	Limited	20			
		G + 72H	0.45			1/1	Limited	20			
	PT Eccentric KF	G + 24H	− 0.68			1/1	Limited	20			
		G + 48H	0.63			1/1	Limited	20			
		G + 72H	0.61			1/1	Limited	20			
	CMJ performance	Post	NS			2/0	No				
		G + 24H	NS			2/0	No				
		G + 48H	0.27			3/1	No	10			
		G + 72H	0.27			3/1	No	10			
	TQR	G + 24H	NS			1/0	No				
		G + 48H	NS			1/0	No				
		G + 72H	NS			1/0	No				

*ṝ*_:_ pooled correlation from Hunter and Schmidt random-effects method, CL: confidence limit, *N*: number of players, TD: total distance, KE: knee extension, KF: knee flexion, CMJ: countermovement jump, CMJ_PPO_: countermovement jump peak power, PT: peak torque, DOMS: delayed onset muscle soreness, BAM+: brief assessment of the mood, HIR: high-intensity running (> 4 m s^−1^), MVIC: maximal voluntary isometric contraction, VHIR: very high-intensity running (> 5.5 m s^−1^), conc: concentric, ecc: eccentric, Acc: acceleration, Decel: deceleration, No: no evidence, NS: not specified, PT 1–100 Hz: peak torque induced by single (1 Hz) and paired electrical stimuli (10 and 100 Hz)

**Table 5:** Summary of findings from meta-analysis describing the relationship between external load metrics (levels 2 and 3) [3] and fatigue-related markers

Predictors	Fatigue-related markers	Time	*ṝ*	Lower CL	Upper CL	Number of studies/correlations	Evidence	*n*	*I*^2^	chi^2^	*p*
HI Acc	Muscle damage	Post	0.24	0.02	0.46	2/1	Limited	45			
		G + 24H	0.41	0.30	0.51	3/5	Moderate	52	0	0.97	0.91
		G + 48H	0.30	0.15	0.45	3/6	Moderate	77	0	2.95	0.71
		G + 72H	0.01			1/1	Limited	10			
	CMJ_PPO_	G + 24H	− 0.61	− 0.79	− 0.42	2/5	Strong	46	24	4	0.41
		G + 48H	− 0.36	− 0.68	− 0.05	2/4	Limited	32	0	3.71	0.29
	MVIC	G + 24H	− 0.79			2/1	Limited	34			
		G + 48H	0.74			2/1	Limited	34			
		G + 72H	0.46			2/1	Limited	34			
	DOMS	G + 24H	− 0.05			3/1	Limited	20			
		G + 48H	0.23	− 0.27	0.73	3/2	Limited	34	79	4.04	0.04
		G + 72H	0.23	− 0.11	0.56	3/2	Limited	34	43	1.84	0.17
	CMJ performance	G + 24H	NS			2/0	No				
		G + 48H	0.27			3/1	Limited	10			
		G + 72H	0.09			1/1	Limited	10			
	Leucocytes count	G + 24H	0.17			1/1	Limited	20			
		G + 48H	− 0.14			1/1	Limited	20			
		G + 72H	− 0.02			1/1	Limited	20			
	PT Concentric KE	G + 24H	− 0.75			1/1	Limited	20			
		G + 48H	0.52			1/1	Limited	20			
		G + 72H	0.56			1/1	Limited	20			
	PT Concentric KF	G + 24H	− 0.84			1/1	Limited	20			
		G + 48H	0.70			1/1	Limited	20			
		G + 72H	0.58			1/1	Limited	20			
	PT Eccentric KE	G + 24H	− 0.64			1/1	Limited	20			
		G + 48H	0.51			1/1	Limited	20			
		G + 72H	0.41			1/1	Limited	20			
	PT Eccentric KF	G + 24H	− 0.89			1/1	Limited	20			
		G + 48H	0.62			1/1	Limited	20			
		G + 72H	0.69			1/1	Limited	20			
	TQR	G + 24H	NS			1/0	No				
		G + 48H	NS			1/0	No				
		G + 72H	NS			1/0	No				
Mod to HI Acc	Muscle damage	Post	0.34			2/1	Limited	35			
		G + 24H	0.32			2/1	Limited	35			
		G + 48H	0.27			2/1	Limited	35			
	CMJ conc force	G + 48H	− 0.49	− 0.78	− 0.17	1/1	Limited	35			
	CMJ ecc force	Post	− 0.47	− 0.73	− 0.11	1/1	Limited	35			
	MVIC	G + 24H	− 0.74			1/1	Limited	35			
		G + 48H	0.80			1/1	Limited	35			
		G + 72H	0.49			1/1	Limited	35			
	DOMS	G + 24H	− 0.04			1/1	Limited	35			
		G + 48H	− 0.02			1/1	Limited	35			
		G + 72H	− 0.04			1/1	Limited	35			
	PT Concentric KE	G + 24H	− 0.75			1/1	Limited	20			
		G + 48H	0.56			1/1	Limited	20			
		G + 72H	0.64			1/1	Limited	20			
	PT Concentric KF	G + 24H	− 0.84			1/1	Limited	20			
		G + 48H	0.70			1/1	Limited	20			
		G + 72H	0.57			1/1	Limited	20			
	PT Eccentric KE	G + 24H	− 0.60			1/1	Limited	20			
		G + 48H	0.57			1/1	Limited	20			
		G + 72H	0.47			1/1	Limited	20			
	PT Eccentric KF	G + 24H	− 0.85			1/1	Limited	20			
		G + 48H	0.65			1/1	Limited	20			
		G + 72H	0.68			1/1	Limited	20			
HI Decel	Muscle damage	Post	0.32	0.13	0.51	3/2	Limited	45			
		G + 24H	0.47	0.36	0.59	5/6	Strong	52	0	1.26	0.87
		G + 48H	0.37	0.18	0.55	6/7	Moderate	67	0	4.8	0.44
		G + 72H	0.00			1/1	Limited	10			
	CMJ_PPO_	G + 24H	− 0.59	− 0.78	0.40	1/4	Limited	32	0	2.49	0.48
		G + 48H	− 0.11	− 0.42	0.19	1/4	Limited	32	0	2.63	0.45
	CMJ performance	G + 24H	NS			1/0	No				
		G + 48H	0.15			2/1	Limited	10			
		G + 72H	0.16			1/1	Limited	10			
	MVIC	G + 24H	− 0.78			2/1	Limited	34			
		G + 48H	0.70			2/1	Limited	34			
		G + 72H	0.46			2/1	Limited	34			
	DOMS	G + 24H	− 0.11			2/1	Limited	34			
		G + 48H	0.08			2/1	Limited	34			
	PT Concentric KE	G + 24H	− 0.82			1/1	Limited	20			
		G + 48H	0.43			1/1	Limited	20			
		G + 72H	0.59			1/1	Limited	20			
	PT Concentric KF	G + 24H	− 0.84			1/1	Limited	20			
		G + 48H	0.70			1/1	Limited	20			
		G + 72H	0.58			1/1	Limited	20			
	PT Eccentric KE	G + 24H	− 0.55			1/1	Limited	20			
		G + 48H	0.53			1/1	Limited	20			
		G + 72H	0.53			1/1	Limited	20			
	PT Eccentric KF	G + 24H	− 0.82			1/1	Limited	20			
		G + 48H	0.62			1/1	Limited	20			
		G + 72H	0.65			1/1	Limited	20			
	Leucocytes count	G + 24H	0.22			1/1	Limited	20			
		G + 48H	0.14			1/1	Limited	20			
		G + 72H	− 0.04			1/1	Limited	20			
	TQR	G + 24H	NS			1/0	No				
		G + 48H	NS			1/0	No				
		G + 72H	NS			1/0	No				
Mod to HI Decel	Muscle damage	Post	0.14			2/1	Limited	35			
		G + 24H	0.03			2/1	Limited	35			
		G + 48H	0.25			2/2	Limited	67			
	MVIC	G + 24H	− 0.80			1/1	Limited	35			
		G + 48H	0.76			1/1	Limited	35			
		G + 72H	0.39			1/1	Limited	35			
	CMJ conc force	G + 48H	− 0.49	− 0.78	− 0.17	1/1	Limited	35			
	CMJ ecc force	Post	− 0.47	− 0.73	− 0.11	1/1	Limited	35			
	DOMS	G + 24H	− 0.14			1/1	Limited	35			
		G + 48H	0.06			1/1	Limited	35			
		G + 72H	0.10			1/1	Limited	35			
	PT Concentric KE	G + 24H	− 0.82			1/1	Limited	20			
		G + 48H	0.40			1/1	Limited	20			
		G + 72H	0.59			1/1	Limited	20			
	PT Concentric KF	G + 24H	− 0.83			1/1	Limited	20			
		G + 48H	0.64			1/1	Limited	20			
		G + 72H	0.57			1/1	Limited	20			
	PT Eccentric KE	G + 24H	− 0.54			1/1	Limited	20			
		G + 48H	0.53			1/1	Limited	20			
		G + 72H	0.42			1/1	Limited	20			
	PT Eccentric KF	G + 24H	− 0.84			1/1	Limited	20			
		G + 48H	0.57			1/1	Limited	20			
		G + 72H	0.71			1/1	Limited	20			
High Impact	Muscle damage	G + 24H	0.19	− 0.14	0.51	2/4	Limited	47	0	3.17	0.37
		G + 48H	0.15	− 0.22	0.53	2/4	Limited	47	0	4.17	0.24
	CMJ_PPO_	G + 24H	− 0.32	− 0.61	− 0.03	2/4	Limited	50	5	5.60	0.23
		G + 48H	0.04	− 0.30	0.38	1/4	Limited	32	0	3.25	0.36

*ṝ*_:_ pooled correlation from Hunter and Schmidt random-effects method, *CL*: confidence limit, *N*: number of players, *TD*: total distance, *KE*: knee extension, *KF*: knee flexion, *CMJ*: countermovement jump, *CMJ*_*PPO*_: countermovement jump peak power, *PT*: peak torque, *DOMS*: delayed onset muscle soreness, *TQR*: total quality of recovery, *BAM+*: brief assessment of the mood, *HI*: high intensity, *Mod*: moderate, *MVIC*: maximal voluntary isometric contraction, *conc*: concentric, *ecc*: eccentric, *Acc*: acceleration, *Decel*: deceleration, *No*: no evidence, *Conf*: conflicted

## Results

### Study and Data Characteristics

**Fig. 1:**
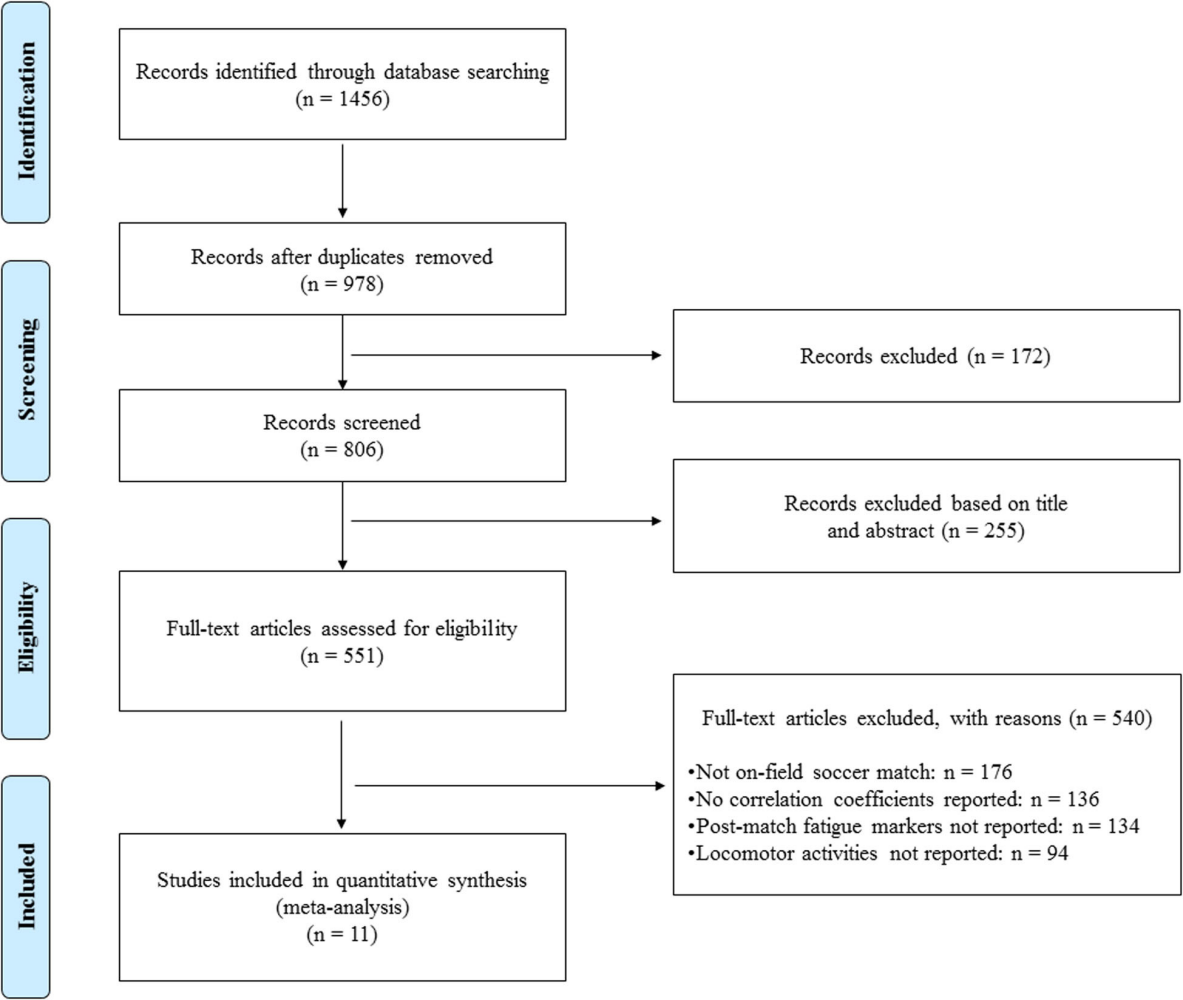
PRISMA flow chart

The flow chart of the search and selection process is presented in Fig. 1. In summary, the searches identified 1456 relevant articles including all reference lists. After critically analyzing the titles and abstracts, the total number of relevant articles was reduced to 551. Once applying the selection criteria, eleven cohort studies were selected (Fig. 1) [12, 22, 23, 25, 36-42] and data were extracted for meta-analysis. The total number of players was 165 with 89% belonging to the elite level and only male soccer players were finally represented (Table 3). The average methodological quality of the included articles was 0.70 ± 0.12 (mean ± standard deviation) out of 1, ranging from 0.40 to 0.85, with 4 articles considered of a high (≥ 0.75) methodological quality (Table 3).

Eight main external load variables with 10 additional derivatives were used in the selected studies as follows:

- Level 1: Total distance (TD, 8 studies), HIR (7 studies), VHIR (7 studies), sprints (3 studies)
- Level 2: Accelerations (5 studies) and decelerations (5 studies), high change of directions (1 study).
- Level 3: High impacts (2 studies)

The most common group of dependent variables measured in the selected studies was the muscle damage biochemical markers (8 studies). The other dependent variables represented in at least two studies were CMJ peak power output (CMJ_PPO_, 4 studies), MVIC (2 studies) and DOMS (2 studies)

These variables were measured within the first 3 h (Post; 6 studies), one (G + 24H 7 studies), two (G + 48H; 8 studies) and three days (G + 72H; 3 studies) post-match.

### Total Distance

In relation to absolute TD, muscle damage markers and CMJ_PPO_ were assessed in at least two studies (*n* = 159 match observations, Table 4). Pooled data showed limited evidence of small (i.e., G + 48H) to moderate (Post and G + 24H) correlations between TD and post-match muscle damage markers (Table 4). The relationships between TD and CMJ_PPO_ were rated as trivial (i.e., G + 24H) to small (i.e., G + 48H). The correlation between relative TD (i.e., TD/min) and CMJ_PPO_ was rated as trivial, similarly to the correlation between relative TD and muscle damage markers (Table 4).

### High-Intensity Running (> 4 m s^−1^):

In relation to HIR, muscle damage markers, DOMS and MVIC were assessed in at least two studies (n = 93 match observations). Pooled data showed strong evidence of a large correlation between HIR and muscle damage at Post ($\overline{r}$ = 0.59; 95% CI = [0.35, 0.84]). At G + 24H and G + 48H, the magnitude of this relationship became moderate with limited evidence (Table 4). Between HIR and MVIC, there was limited evidence of a negative and large correlation at G + 24H and positively moderate correlations at G + 48H and G + 72H. The relationship between HIR and DOMS was rated as trivial at all time points (Table 4).

### Very High-Intensity Running (> 5.5 m s^−1^):

In relation to absolute VHIR, muscle damage markers and CMJ_PPO_ were assessed in at least two studies. Pooled data (*n* = 146 match observations) showed moderate to strong evidence of large correlations between VHIR and muscle damage at Post ($\overline{r}$ = 0.67; 95% CI = [0.40, 0.94]) and at G + 24H ($\overline{r}$ = 0.54; 95% CI = [0.35, 0.65]). At G + 48H, the magnitude of this relationship became moderate with limited evidence. Between VHIR and CMJ_PPO_, there was strong evidence of a negative and large pooled correlation (ṝ = − 0.52; 95% CI = [− 0.64, − 0.40]) at G + 24H. This relationship was rated as moderate at G + 48H with conflicted evidence (Table 4).

The correlations between relative VHIR (per unit of time) and muscle damage markers and CMJ_PPO_ output were rated as small, with limited evidence. Relative VHIR was moderately (G + 24H) to largely (G + 48H) correlated with the brief assessment of mood (1 study, *n* = 35), with limited evidence (Table 4).

### Sprint Running

Muscle damage markers and MVIC were assessed with sprint running performance during the match. Data showed moderate to large correlations between sprint running distance and these two fatigue-related markers from G + 24H to G + 72H, with limited evidence (Table 4).

### Acceleration Variables

Acceleration variables were related to muscle damage markers, CMJ_PPO_, MVIC, and DOMS. Pooled data showed strong (i.e., at G + 24H) and limited (at Post and G + 48H) evidence of moderate correlations between high accelerations and muscle damage markers (Table 5).

There was moderate evidence of a large and negative correlation ($\overline{r}$ = − 0.61; 95% CI = [− 0.82, − 0.42]) at G + 24H between CMJ_PPO_ and high accelerations (Table 5). At G + 48H, this relationship was rated as moderate with limited evidence. There was limited evidence of moderate (i.e., G + 72H) to very large (G + 24H and G + 48H) correlations between high accelerations and MVIC of the dominant leg (Table 5). DOMS presented trivial to small correlations with high accelerations regardless of the time points.

### Deceleration Variables

Deceleration variables were related to muscle damage markers, CMJ_PPO_ and DOMS. Pooled data (3 studies, *n* = 70 match observations) showed limited (at Post and G + 48H) and moderate (i.e., at G + 24H) evidence of positive and moderate correlations between high decelerations and muscle damage markers. There was limited evidence of small (G + 48H) to large (G + 24H) correlations between high decelerations and CMJ_PPO_. DOMS presented trivial correlations with high decelerations regardless of the time points (Table 5).

### High Impacts

In relation to the number of high impacts, muscle damage markers and CMJ_PPO_ were assessed. Pooled data (2 studies, *n* = 40 match observations) showed conflicted evidence of small correlations between high impact and muscle damage markers at G + 24H and G + 48H. There was also conflicted evidence for the moderate correlation between high impacts and CMJ_PPO_ at G + 24H (Table 5).

### Regression Analysis

**Fig. 2:**
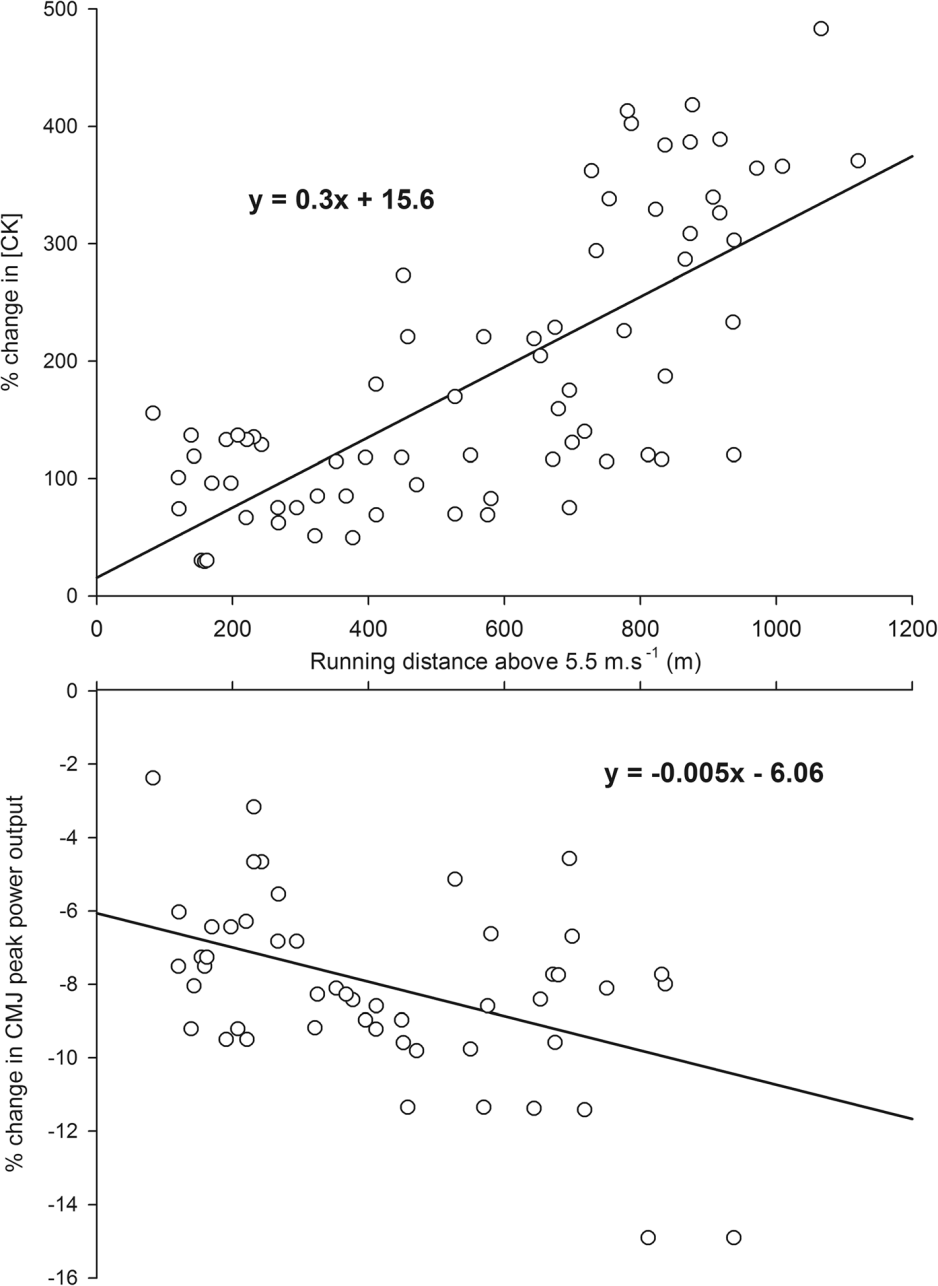
Relationship between match-related running distance above 5.5 m s^−1^ and post-match changes in CK (upper panel) and CMJ peak power output (lower panel). CK: creatine kinase; CMJ: countermovement jump

The relationship between VHIR and fatigue-related markers (percentage change in CMJ_PPO_ and CK, Fig. 2) could be represented by a linear function (3 studies, 2 of high quality, 11 matches, *n* = 87 player-match observations) (Fig. 2).

## Discussion

The aim of this systematic review with meta-analysis was to examine whether external load metrics during soccer match-play reflect acute and residual changes in post-match biochemical, neuromuscular, and perceptual responses. The main findings were as follows: (1) the match-related running distance above 5.5 m s^−1^ was identified as the only monitoring variable largely correlated with both biochemical and neuromuscular markers, (2) practically, at G + 24H, for each 100 m of VHI running distance covered, CK activity would increase by 30% and CMJ_PPO_ would decrease by 0.5%.

Among the biochemical variables systematically reviewed, there was strong evidence of two large pooled correlations (*r* = 0.54 to 0.67) between HIR or VHIR and changes in muscle damage markers (e.g., CK) at Post and G + 24H, respectively. These changes were also moderately correlated with high accelerations at G + 24H. The weaker correlation may be due to the restricted number of selected studies examining acceleration variables. Over fifty listed variables, high accelerations (rank four) and VHIR (rank two) were reported as the monitored variables that were the most used by elite soccer team practitioners to quantify training and match loads [8]. From an injury prevention standpoint, hamstring strain injuries predominantly occur during VHIR and high acceleration [20, 42, 43]. Additionally, injury to the hamstrings muscle group is the most commonly reported injury in male soccer players [42, 44, 45]. Therefore, in the selected studies, the MVIC evaluation was focused on the hamstrings muscles (i.e., at G + 24H and G + 48H) [21, 25]. Indeed, the injury mechanism may be due to the repeated and excessive lengthening demands placed on the hamstrings during the high eccentric force contractions involved during these specific efforts (e.g., VHIR) [43, 46]. Furthermore, as also supported by our results (Tables 4 and 5), exercise-induced muscle damage is more typically associated with the performance of fast eccentric muscle actions (e.g., high accelerations) than to the execution of lower velocity-based eccentric muscle actions (e.g., moderate accelerations) [47]. These fast eccentric muscle actions would exacerbate the mechanical stress characterized by cellular and subcellular structural disturbances, such as the focal disruption of the myofibers and cytoskeleton resulting in z-disk streaming [48, 49]. From our results, VHIR may represent the most sensitive external load metric to monitor changes in acute (immediately post-match) and residual (i.e., at G + 24H only) muscle damage status. Interestingly, while cumulative exposure or large week-to-week changes in VHIR may represent a substantial increase in injury risk [50–52], a high but gradual exposure to VHIR may confer additional protection to spikes in workload for soccer players [53].

While practitioners have reported the total distance covered as the most commonly tracked variable [8], our results show no evidence of any significant relationship with changes in muscle damage markers. In contrast to VHIR, low to moderate running intensities are thought to induce a lower magnitude of muscle damage [54], without significant perturbation in the membrane permeability [38, 55]. As a large proportion of the match-related total distance is covered at low intensity (e.g., walking, jogging) [56], this may explain an absence of a relationship with changes in muscle damage markers. Additionally, total distance has been largely correlated with salivary cortisol concentrations at G + 24H and G + 48H in only one study (Tables 4 and 5) [57]. These relationships with residual endocrine responses would need to be confirmed by future investigations.

Our results displayed a large correlation (strong evidence) between VHIR and change in CMJ_PPO_, yet at G + 24H only. This change in CMJ_PPO_ was largely correlated with high accelerations at the same time-point but with moderate evidence due to the restricted number of studies considering this metric. These results highlight that the reduction in CMJ_PPO_ at G + 24H, in addition to the increase in muscle damage markers, may be related to the repetitive stress (i.e., mechanical) sustained by the neuromuscular system throughout the frequent VHIR and high acceleration actions [58]. The amount of eccentric-related actions likely characterizes this mechanical stress potentially inducing changes in joint sequencing (e.g., increase in eccentric phase duration), in a motor pattern used for performance (adjustment of neuromuscular recruitment strategies) [59, 60] and selective damage of type II muscle fibers [61]. Accordingly, it has been recently determined that an increase by 0.6 km in VHIR, as a specific match-related intense activity, may induce a decrement in CMJ_PPO_ by slightly more than the smallest worthwhile change (i.e., 1.0 W/kg) [62]. Consequently, the present meta-analytic impairment in CMJ_PPO_ may reflect the power-based load characterized by VHIR during soccer matches. Conversely, total distance covered, including a low proportion of high-intensity eccentric actions, was not related to post-match changes in CMJ_PPO_.

Our results do not demonstrate evidence for significant relationships between any tracking variable and changes in post-match perceptual responses. While self-report, perceptual measures such as questionnaires are simple and efficient methods [63] to assess match-related load, only a few studies have investigated such a relationship [21, 25, 32]. Today, a large majority of elite soccer clubs collect self-report measures (e.g., perceived recovery such as TQR) daily to monitor players’ training-induced psychometric and wellbeing states [8]. However, they do not seem to use this monitoring tool as frequently for reflecting fatigue associated with performing home and away matches. This seems surprising since the match load represents the main determinant of a high weekly training load during the competitive season [64]. Particularly, subjective measures of mood disturbance, perceived stress and recovery may reflect acute and chronic loads with superior sensitivity and consistency in reference to more objective measures (e.g., muscle damage markers, CMJ_PPO_) [65]. In our study, the lack of an association between subjective and objective measures provides support for the complementary inclusion of both measurements.

Practically, at G + 24H, for every 100 m of VHIR during a soccer match, CK activity would increase by 30% and CMJ_PPO_ would decrease by 0.5% (Fig. 2). Our meta-analytic results show, for the first time, that VHIR appears as the strongest predictor of alterations in muscle damage and peak power output since it was the only tracking variable largely related to these biochemical and neuromuscular fatigue-related makers. The systematic and meta-analysis of the current literature suggests that the running distance covered above 5.5 m s^−1^, may explain up to ~50% of the biochemical and neuromuscular post-match states. To date, other significant relationships between external load and post-match monitoring variables have yet to be determined for residual fatigue status especially at G + 48H and G + 72H.

The strongest correlations between external load metrics (i.e., VHIR) and post-match muscle damage or CMJ_PPO_ have been reported for acute fatigue (i.e., at Post) and at G + 24H only, when magnitude of changes in most of the fatigue-related markers are at their greatest (i.e., peak changes) [10, 13, 55]. There was no evidence of any significant relationships at G + 48H and G + 72H. The restricted number of studies (10 studies) examining the multi-factorial nature of fatigue incurred post-soccer match limits the strength of some of our conclusions. Furthermore, given the complexity of fatigue causing mechanisms and those responsible for its reversal, biochemical and neuromuscular changes induced by a match exhibit considerable variability [10, 55, 66]. Regarding muscle damage markers, and more specifically CK, part of the variability may be attributed to their rate of clearance from the circulation [67]. In addition, the players’ aerobic fitness level [21] and specific neuromuscular characteristics (e.g., lower body strength) [68–71] have been associated with match-related activity and fatigue development. Additionally, players’ degree of familiarization to eccentric training and actions (e.g., high decelerations) are believed to impact their recovery rate [72]. All these between-player discrepancies may explain weak correlations between some external load metrics (e.g., VHIR and/or acceleration patterns) and fatigue-related markers at G + 48H and G + 72H.

One limitation of this present review with meta-analysis is the between-study differences in definitions regarding the main players’ external load metrics. Indeed, while most of the selected studies tracked match-related sprinting efforts, only two of them applied a speed threshold above 25 km h^−1^, leading to a weaker strength of findings. As previously highlighted by others, gathering external load data from different tracking technologies and different products may induce some flaws (e.g., no agreed filtering methods, sampling rates, and data-processing algorithms across studies). As example, there can be substantial differences between products, particularly for threshold-based acceleration and deceleration variables [73, 74]. Finally, a relatively small number of studies was included in our analysis. Remarkably, all selected studies have been published over the last 7 years due to the recent technology development that allows collecting locomotor-related activities during competition. Additionally, these technologies are either mainly restricted to home matches (i.e., semi-automatic cameras or radio-frequency systems) or not allowed during official matches (i.e., GPS), while the situation is now evolving. Considering these limitations, further investigations would be needed to ascertain the strength of evidence regarding sprint (> 7 m s^−1^), acceleration and deceleration variables. Moreover, further studies should investigate the use of individualized external load thresholds (based on players’ physiological characteristics such as maximal aerobic speed and sprinting speed )[75, 76] can more efficiently reflect the acute and residual changes in post-match muscle damage, neuromuscular and perceptual responses.

## Conclusions

While total distance is likely the most commonly monitored variable in elite soccer, it is not associated with changes in any post-game fatigue-related markers. A unique finding of our meta-analysis, however, was the observation of large correlations between match-related VHIR (above 5.5 m s^−1^) distance and both acute (Post) and residual (G + 24H but not G + 48H/G + 72H) changes in fatigue-related markers. Indeed, VHIR was identified as the only tracking variable that correlated largely with both biochemical and neuromuscular markers. Practically, at G + 24H, for every 100 m of VHI running distance covered, CK activity would increase by 30% and CMJ_PPO_ would decrease by 0.5%. VHIR, at least when assessed during the first 24 h of the recovery process, represents the most sensitive tracking variable to depict biochemical and neuromuscular loads resulting from soccer match-play.

## Data Availability

The datasets used and/or analyzed during the current study are available from the corresponding author on reasonable request.

## Abbreviations

Acc: Acceleration
BAM+: Brief assessment of the mood
CK: Creatine kinase
CMJ: Countermovement jump
CMJ_PPO_: Countermovement jump peak power output
conc: Concentric
Decel: Deceleration
DOMS: Delayed onset muscle soreness
ecc: Eccentric
GPS: Global positioning system
HIR: High-intensity running distance
KE: Knee extension
KF: Knee flexion
G + 24H: Measured 24 h the match
G + 48H: Measured 48 h after the match
G + 72H: Measured 72 h after the match
Mod: Moderate intensity running
MVIC: Maximal voluntary isometric contraction
Post: Measured after the match
PT: Peak torque
TD: Total distance covered
TQR: Total quality of recovery
VHIR: Very high-intensity running distance
